# The role of neutrophils in corneal nerve regeneration

**DOI:** 10.1186/s12886-023-03088-9

**Published:** 2023-07-28

**Authors:** Xiaowen Zhao, Minghong Zhang, Fengjiao Li, Cuiping Ma, Dianqiang Wang, Ye Wang

**Affiliations:** 1 Core Laboratory, The Affiliated Qingdao Central Hospital of Qingdao University, No. 127th, South Siliu Road, Qingdao, Shandong 266042 China; 2 Qingdao Aier Eye Hospital, No. 519th, Zhujiang Road, Qingdao, Qingdao, Shandong 266500 China; 3 Department of Opthalmology, Shandong Provincial Hospital Affiliated to Shandong First Medical University, No. 324th, Jing wu wei qi Road, Jinan, Shandong 250021 China; 4 Shandong Provincial Key Laboratory of Biochemical Engineering, Qingdao Nucleic Acid Rapid Detection Engineering Research Center, College of Marine Science and Biological Engineering, Qingdao University of Science and Technology, No. 54th, Zhengzhou, Road, Qingdao, Shandong 266042 China

**Keywords:** Corneal nerve, Corneal scraping, Neutrophils, *MMP9*, *EGF*

## Abstract

**Background:**

To investigate the role of neutrophils in corneal nerve regeneration.

**Methods:**

A mouse model simulating corneal nerve injury was established and samples from corneal scraping with and without neutrophil closure were collected. These samples were used for corneal nerve staining, ribonucleic acid sequencing, and bioinformatics. Differential expression analysis was used to perform enrichment analysis to identify any significant differences between these two groups. The differential genes were then intersected with neutrophil-associated genes and a protein-protein interaction network was constructed using the intersected genes. The immune infiltration between the two groups was examined along with the immune cell variation between the high and low gene expression groups.

**Results:**

Neutrophil removal delays corneal epithelial and nerve regeneration. A total of 546 differential genes and 980 neutrophil-associated genes, with 27 genes common to both sets were obtained. Molecular Complex Detection analysis yielded five key genes, namely integrin subunit beta 2 (*ITGB2*), matrix metallopeptidase 9 (*MMP9*), epidermal growth factor (*EGF*), serpin family E member 1 (*SERPINE1*), and plasminogen activator urokinase receptor (*PLAUR*). Among these genes, *ITGB2*, *SERPINE1*, and *PLAUR* exhibited increased expression in the neutrophil-confined group, while *MMP9* and *EGF* showed decreased expression, with *MMP9* and *EGF* displaying a more significant difference. Immune infiltration was also observed between the two groups, revealing significant differences in the infiltration of M0 macrophages, activated mast cells, and neutrophils. Moreover, the neutrophil levels were lower in the groups with low *MMP9* and *EGF* expressions and higher in the high-expression group.

**Conclusion:**

Neutrophil confinement might significantly affect the *MMP9* and *EGF* expression levels. Strategies to inhibit *MMP9* could potentially yield therapeutic benefits.

## Introduction

The cornea, in its normal state, possesses a dense network of nerve endings that play a crucial role in sensing and transmitting external stimuli, including tactile, temperature, chemical, and mechanical stimuli. These corneal nerves primarily comprise sensory nerve fibers, along with a small number of sympathetic and parasympathetic nerve fibers. Originating from the ophthalmic branch of the trigeminal nerve, the sensory nerves establish a unique epithelial–neural connection-like structure with the corneal epithelium, which is distributed among the superficial epithelial cells [1]. This normal innervation within the corneal epithelium is important in preserving its integrity, corneal sensitivity, and immune properties, all while functioning in the absence of blood vessels.

Corneal nerves possess a certain degree of regenerative capacity. Clinical confocal observations revealed that 94% of patients with myopia exhibited regrowth of corneal nerves into the corneal flap within a month after undergoing excimer laser in situ keratomileuses, while 23% of patients displayed corneal nerve regeneration within a 3 mm range from the central cornea 3 months after surgery. Furthermore, the successful restoration of the ocular surface’s physiological balance after surgery was closely associated with the regeneration of corneal nerves and the expression of corneal neurotrophic factors [2]. Corneal limbal stem cells can mediate corneal epithelial regeneration [3, 4]. Corneal epithelial wound healing involves various cellular events, such as migration, proliferation, adhesion, and differentiation, all of which are regulated by various molecular signals, including growth factors, cytokines, and neuropeptides [5]. However, the occurrence of inflammatory and autoimmune diseases can impede wound healing [6].

In contrast, neutrophils are the first inflammatory cells to infiltrate the wound. These cells are derived from bone marrow stem cells and constitute approximately 50–70% of peripheral blood leukocytes [7]. Neutrophils are often considered important killer cells that mediate the development of multiple inflammatory conditions and diseases through direct phagocytosis of pathogens and the formation of neutrophil extracellular traps after their necrosis or apoptosis [8, 9]. However, recent studies have revealed an active role of neutrophils in wound healing. When the cornea sustains damage, large numbers of neutrophils converge at the wound site within 6 h of injury. They mediate the post-injury inflammatory response and participate in regulating corneal epithelial healing. Moreover, studies have demonstrated that neutrophils and their lysates significantly impede wound healing in mouse corneal epithelial cells in vitro [10]. Nevertheless, our current understanding of the underlying mechanisms governing neutrophil-mediated corneal injury or healing responses remains incomplete. Therefore, this study aimed to investigate the mechanisms underlying the actions of neutrophils in corneal scraping.

## Methods

### Animals

Male C57BL/6 mice, aged 6–8 weeks, were procured from Beijing Huafeng Biotechnology Co. The animal experiments were approved by the Institutional Animal Care and Use Committee, Qingdao University (Qingdao, Shandong, China). All procedures involving the animals adhered to the guidelines outlined by the Association for Research and the Vision and Ophthalmology statement regarding the use of animals in ophthalmic and vision research. The study is reported in accordance with Animal Research: Reporting of In Vivo Experiments guidelines (https://arriveguidelines.org).

### Neutrophil clearance model construction

Neutrophil-cleaved C57BL/6 mice were intraperitoneally injected with the corresponding immune cell clearance reagent Anti-Gr-1 (200 µl/dose) (Ly6G blocking: 108,453 Biolegend, USA) 24 h before, immediately after, and 24 h after surgery. In contrast, normal C57BL/6 mice were injected with an equivalent volume of phosphate-buffered saline (PBS).

### Corneal epithelial injury model construction

Neutrophil-cleared mice and age-matched normal mice (6–8 weeks) were anesthetized with an intraperitoneal injection of chlorpromazine hydrochloride (7 mg/kg) and ketamine (70 mg/kg). Local anesthesia was then applied to the cornea using proparacaine hydrochloride eye drops (Algernon). A 2-mm diameter corneal ring drill was carefully positioned on the central cornea and rotated perpendicularly for 1 week. Subsequently, the central corneal epithelium (2 mm in diameter) of the mice was scraped using the Algerbrush II corneal rust remover. Oxyfluoxacin eye drops were administered to prevent infection. Corneal epithelial defects were assessed at 0, 24, and 48 h using 0.25% sodium fluorescein and photographed under a BQ900 slit lamp (Haag-Streit, Berne, Switzerland). The stained area was analyzed using Image J software, and the percentage of residual epithelial defects was calculated.

### Corneal nerve staining

Corneal samples obtained from each mouse were fixed using 4% paraformaldehyde and stained with anti-b III tubulin antibodies (Anti-Beta III tubulin antibodies: Biolegend 657,403). Representative images of the stained samples were captured using a Nikon Eclipse Ni-U fluorescence microscope (Nikon, Tokyo, Japan). The corneal nerves were quantified using ImageJ software (National Institutes of Health, Bethesda, Maryland).

### Differential analysis

The limma package in Statistical R was used to identify the differentially expressed genes between corneal scrapings with and without neutrophilic closure. A cut-off value of p < 0.05 and |log2(FC)| > 1 was employed.

### Gene Ontology (GO) annotation and Kyoto Encyclopedia of genes and genomes (KEGG) analysis

The differentially expressed genes were subjected to GO and KEGG enrichment analyses using the ClusterProfiler R package. The results were mapped using the ggplot2 R package. The GO annotations encompassed three categories: biological process, cellular component, and molecular function. Statistical significance was set at p < 0.05.

### Gene set enrichment analysis (GSEA)

This analysis was performed using the enrichplot R package to estimate the significance of the enrichment score (ES). The ES was used to determine the biological significance of the gene set. Multiple hypothesis tests were conducted to calculate the false-positive discovery rate (FDR), with screening criteria set as FDR < 0.25, p < 0.05, and normalized ES (NES) > 1 or < -1.

### Protein-protein interaction (PPI) network construction

The PPI network was constructed using the STRING online tool (https://cn.string-db.org/). Subsequently, the network was imported into the Cytoscape software (https://cytoscape.org/) for visualization, and the modules were completed using the Molecular Complex Detection (MCODE) algorithm within Cytoscape.

### Neutrophil gene origin and immune infiltration

Genes associated with the neutrophil biological function were obtained from the GSEA online database (https://www.gsea-msigdb.org/gsea/index.jsp). The immune infiltration level in each sample was analyzed using the ImmuCellA online tool (http://bioinfo.life.hust.edu.cn/ImmuCellAI#!/analysis).

### Statistical analysis and mapping

The calculations were performed, the images were plotted using GraphPad Prism8, and comparisons between the two groups were made using the t-test. Correlation analysis was performed using the Pearson R test. Statistical significance was set at p < 0.05.

## Results

### Removal of neutrophils delays corneal epithelial and nerve regeneration

**Fig. 1:**
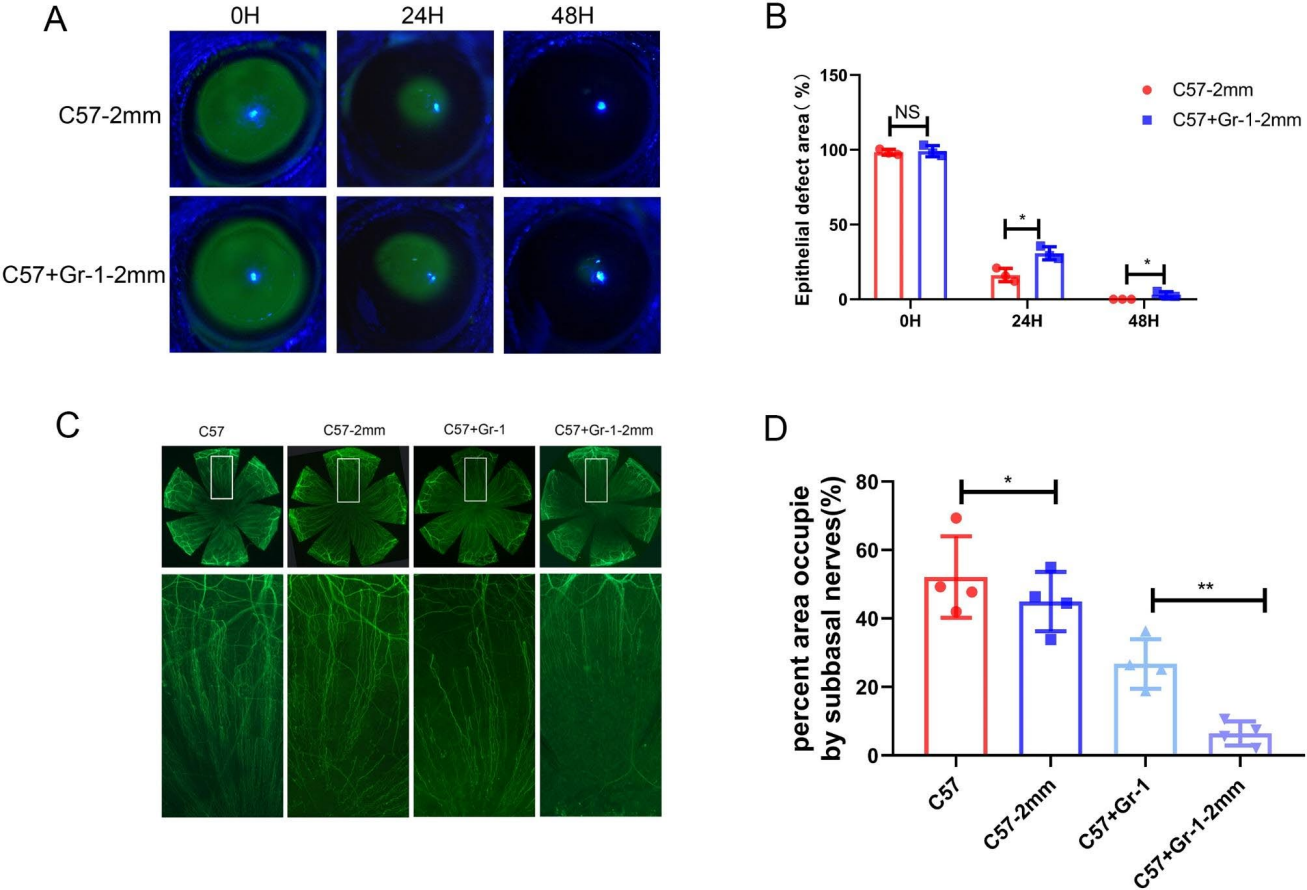
Neutrophil clearance delays regeneration of corneal epithelium and nerves. Neutrophil-cleared mice and control mice treated with 2 mm central corneal epithelial debridement according to the method described above. (**A**, **B**) The exfoliated area and healing process were observed by fluorescein staining using Image J software for analysis (n = 4). (**C-D**) Corneas were collected 48 h post-injury for whole corneal β tubulin III staining (n = 4). Data is represented as mean ± standard deviation. * p < 0.05; ** p < 0.01

Neutrophils promote corneal epithelial and nerve regeneration through the phagocytosis of injury-induced cellular debris and the secretion of cytokines and neurotrophic factors. Age-matched normal mice with or without neutrophil clearance models were evaluated to investigate the mechanism of neutrophil action on corneal epithelial wound healing. Significant differences were observed in corneal re-epithelialization after 2 mm central corneal epithelial scraping (Fig. 1A). Corneal epithelial defects were significantly delayed in neutrophil-cleared mice (24 h: 30.8 ± 4.3%, 48 h: 3.1 ± 1.9%) compared with the control mice (24 h: 16.2 ± 4.4%, 48 h: 0%) (Fig. 1B). Similarly, neutrophil removal resulted in the regression of corneal nerves and corneal nerve regeneration (Fig. 1C). Further, corneal nerves regressed significantly in neutrophil-cleared mice (48 h: 54.6 ± 10.5%) compared with the control mice (48 h: 26.8 ± 7.2%). Forty-eight hours after corneal epithelial injury, neutrophil-cleared mice (6.4 ± 5.3%) exhibited a significant delay in corneal nerve regeneration compared with the control mice (39.9 ± 7.2%) (Fig. 1D). Image J software (n = 4 per group) was employed to analyze the corneal injury area and corneal nerve density, and the data is represented as the mean ± standard deviation. * p < 0.05, ** p < 0.01.

### Analysis of variance

**Fig. 2:**
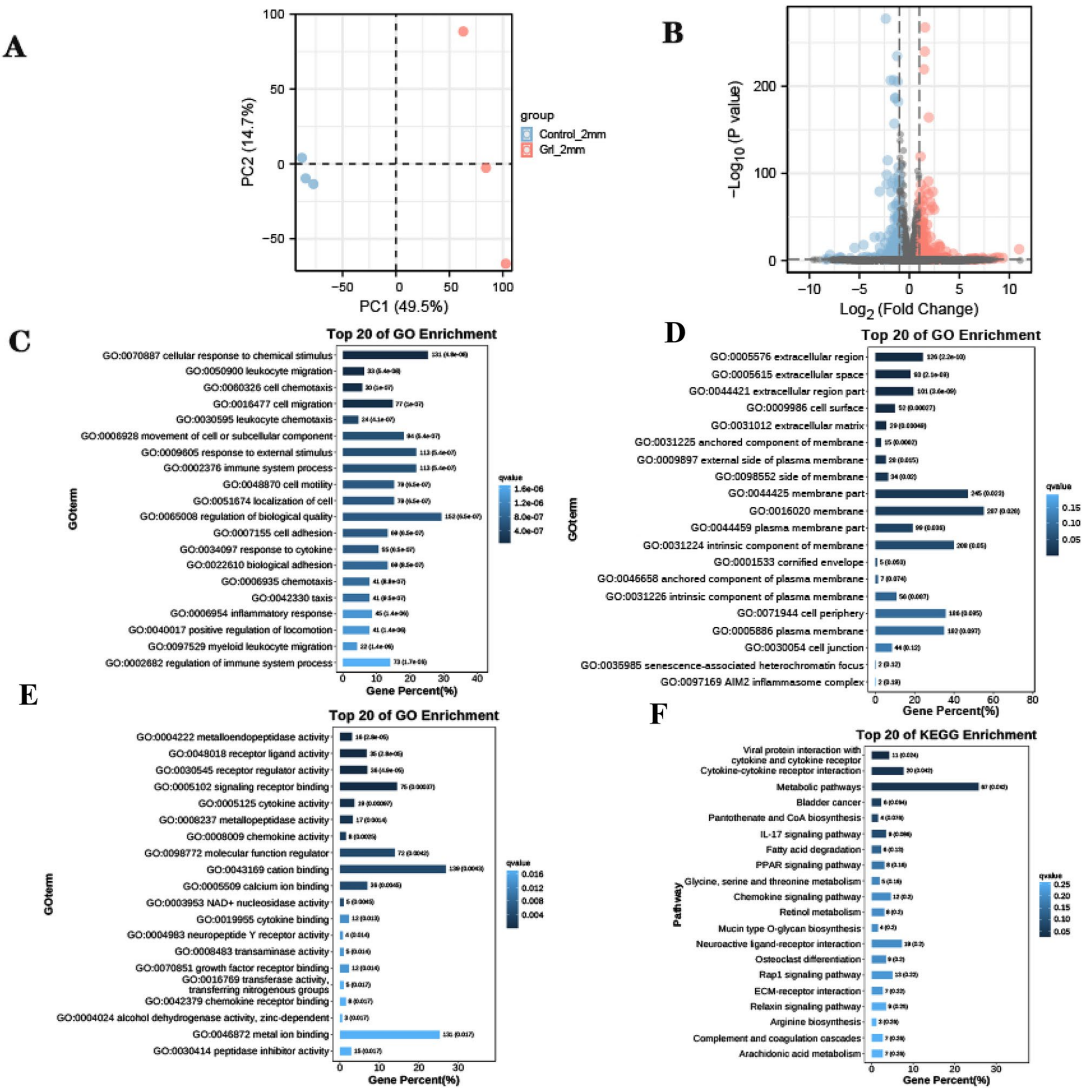
**Differential gene screening.** (**A**) PCA analysis showing sample composition. (**B**) Differential volcano plot, blue indicates down-regulated genes, red indicates up-regulated genes. Gene Ontology analysis of differentially expressed mRNAs. (**C**) Biological process (BP) was evaluated separately and the top 20 significant terms of all ontologies are shown. (**D**) Cellular component (CC) was evaluated separately and the top 20 significant terms of all ontologies are shown. (**E**) Molecular function (MF) was evaluated separately and the top 20 significant terms of all ontologies are shown. (**F**) Top 20 Kyoto Encyclopedia Genes and Genomes (KEGG) pathways was shown

Initially, a differential expression analysis was conducted to compare corneal scraping samples with and without neutrophil closure. The screening criteria included a threshold of |log2(FC)| > 1 and p < 0.05. A total of 546 differentially expressed genes were screened, of which 316 were up-regulated genes and 230 were down-regulated genes. Subsequently, to gain insight into the biological roles of the most significantly down- or upregulated differentially expressed genes, GO and KEGG analyses were performed. Figure 2 shows the GO and KEGG analysis of differentially expressed mRNAs. The three GO classifications of biological processes (BPs), cellular components (CCs), and molecular functions (MFs) were identified and evaluated separately. The GO analysis results revealed significant enrichment of the differential genes in cellular responses to chemical stimuli, leukocyte migration, cell chemotaxis, cell migration, leukocyte chemotaxis, and leukocyte chemotaxis. Additionally, the KEGG analysis revealed significant enrichment of the differential genes in viral protein interactions with cytokines and cytokine receptors, interactions between cytokines and cytokine receptors, and metabolic pathways.

### GSEA

**Fig. 3:**
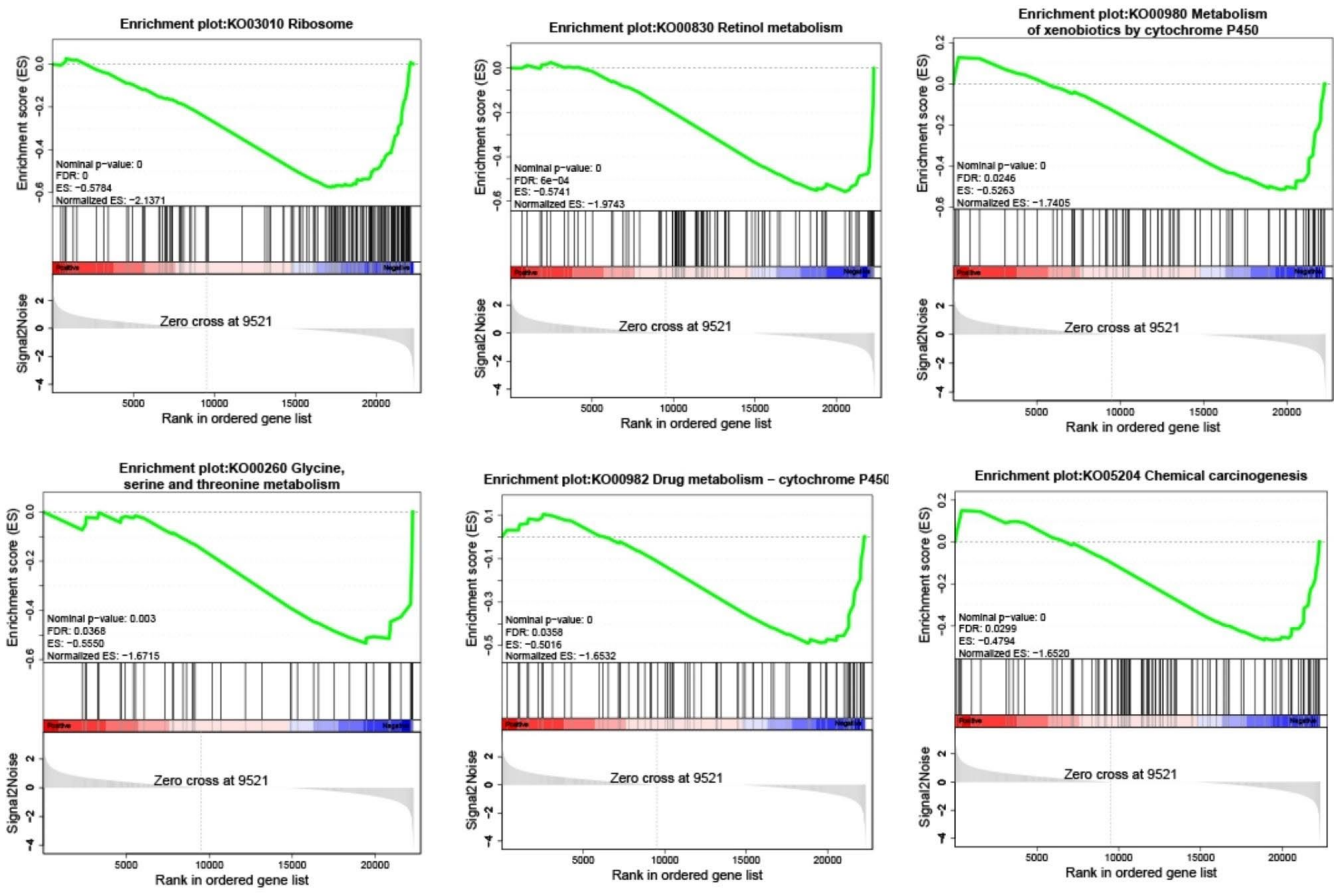
GSEA analysis shows the first six pathways that are significantly enriched

GSEA was performed with the inclusion of two subgroups, namely neutrophil confinement and no neutrophil confinement. The screening criteria employed were |NES| > 1, FDR < 0.25, and p < 0.05. The results revealed notable differences between the two groups. Specifically, the neutrophil confinement group exhibited higher levels of ribosome metabolism, retinol metabolism, cytochrome metabolism of toxic substances, glycine, serine, and threonine metabolism, drug metabolism–cytochrome, and chemical carcinogenicity pathways when compared with the no-neutrophil confinement group, as presented in Fig. 3.

### Genes associated with neutrophil biological functions

**Fig. 4:**
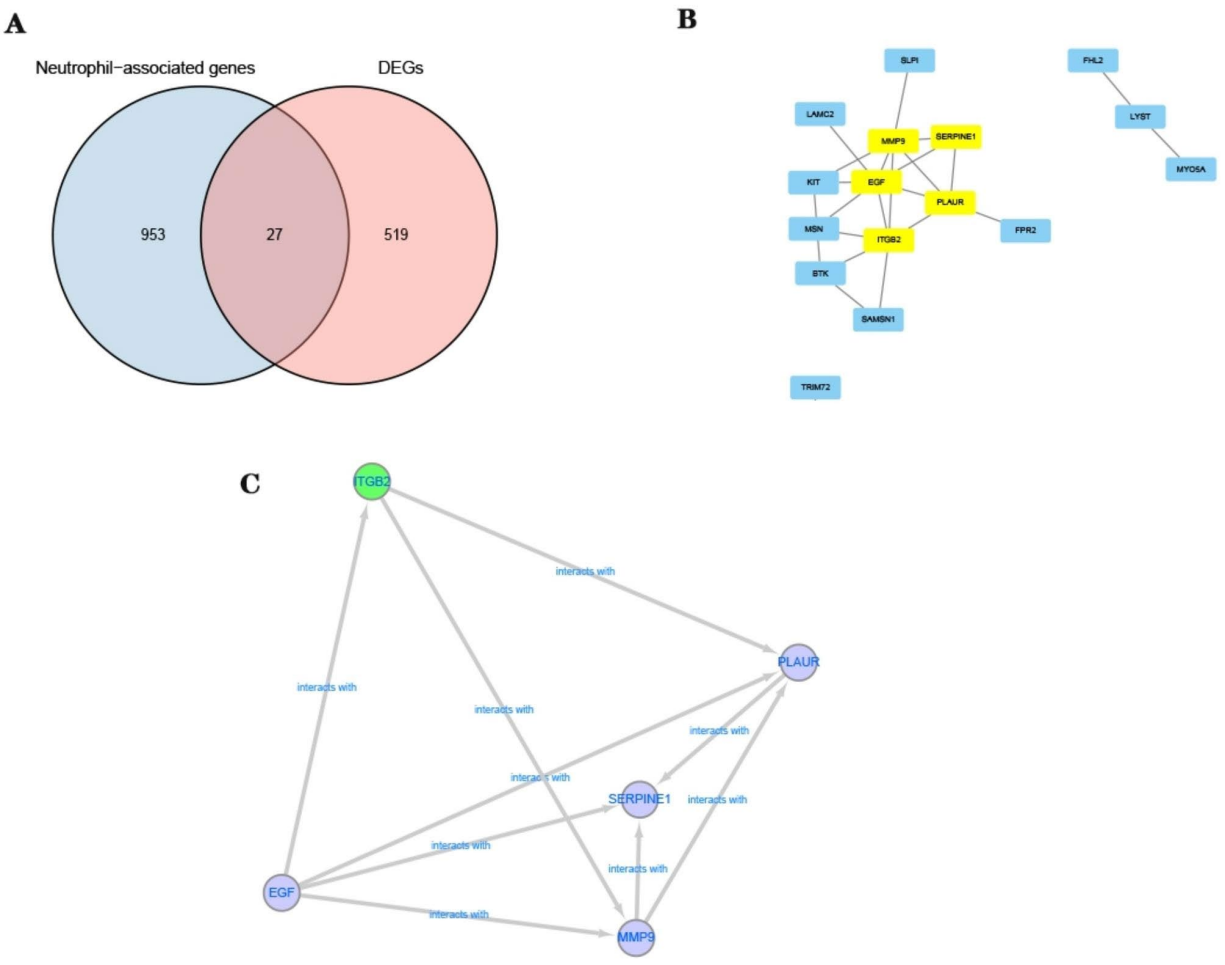
Genes associated with neutrophils. (**A**) Venn diagram showing common genes associated with both neutrophils and differential genes. (**B**) PPI network diagram. (**C**) 5 key genes

A gene set was compiled and intersected with differential genes to identify genes associated with neutrophil biological functions, resulting in intersecting genes. Subsequently, a PPI network was constructed using these intersecting genes and screened key genes. Five key genes, namely integrin subunit beta 2 (*ITGB2*), matrix metallopeptidase 9 (*MMP9*), epidermal growth factor (*EGF*), serpin family E member 1 (*SERPINE1*), and plasminogen activator urokinase receptor (*PLAUR*), were screened for analysis (Fig. 4).

### Expression observation

**Fig. 5:**
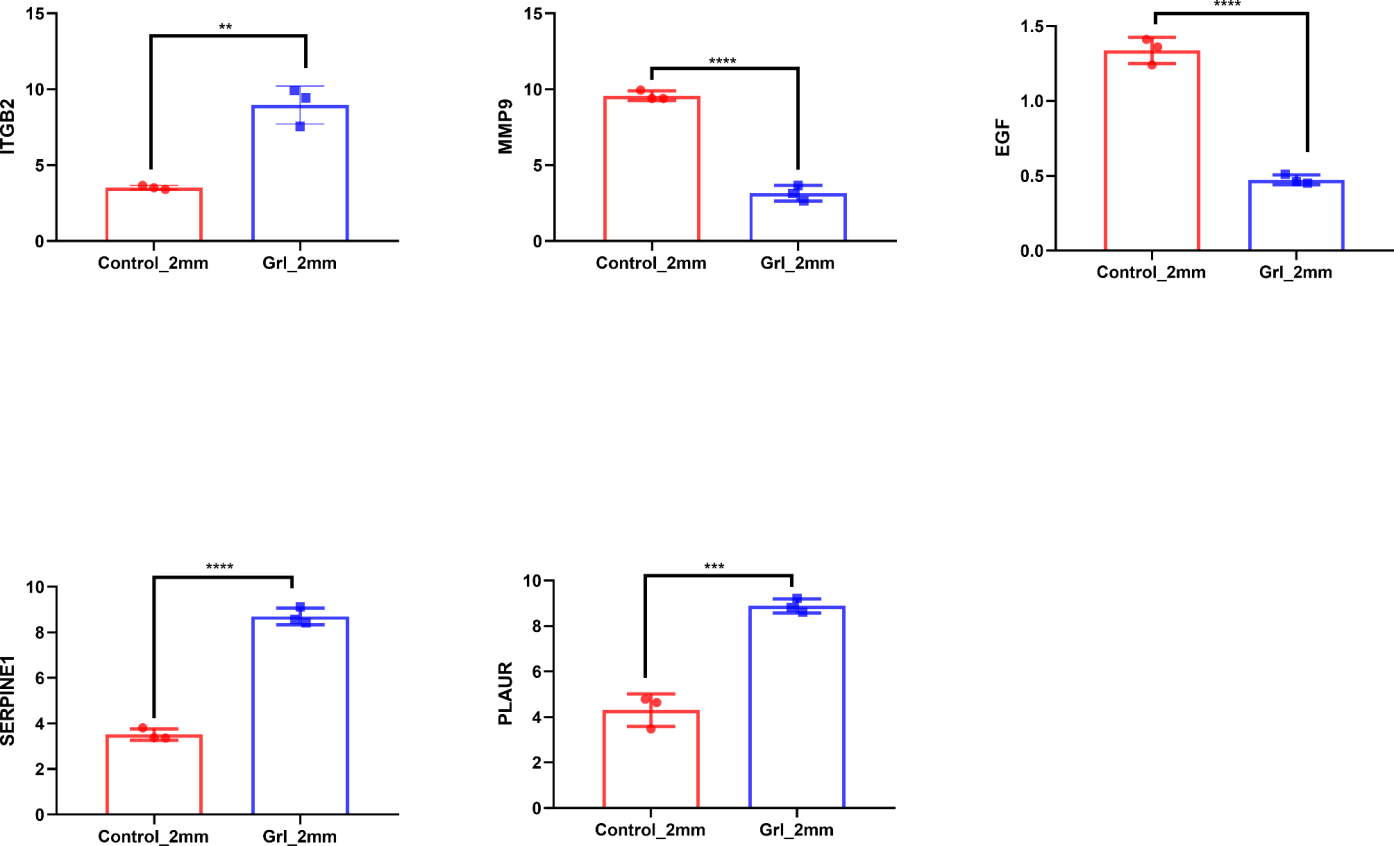
Expression of ITGB2, MMP9, EGF, SERPINE1, PLAUR in the presence and absence of neutrophil closure. **p < 0.01; ***p < 0.001; ****p < 0.0001

Subsequently, the *ITGB2*, *MMP9*, *EGF*, *SERPINE1*, and *PLAUR* expressions were examined in the presence and absence of neutrophil closure. The findings revealed that *ITGB2*, *SERPINE1*, and *PLAUR* exhibited higher expression levels in the neutrophil-confined group compared with the unneutrophil-confined group. Conversely, *MMP9* and *EGF* exhibited lower expression levels in the neutrophil-confined group. The results are presented in Fig. 5.

### Expression correlation of genes with other central nodes

**Fig. 6:**
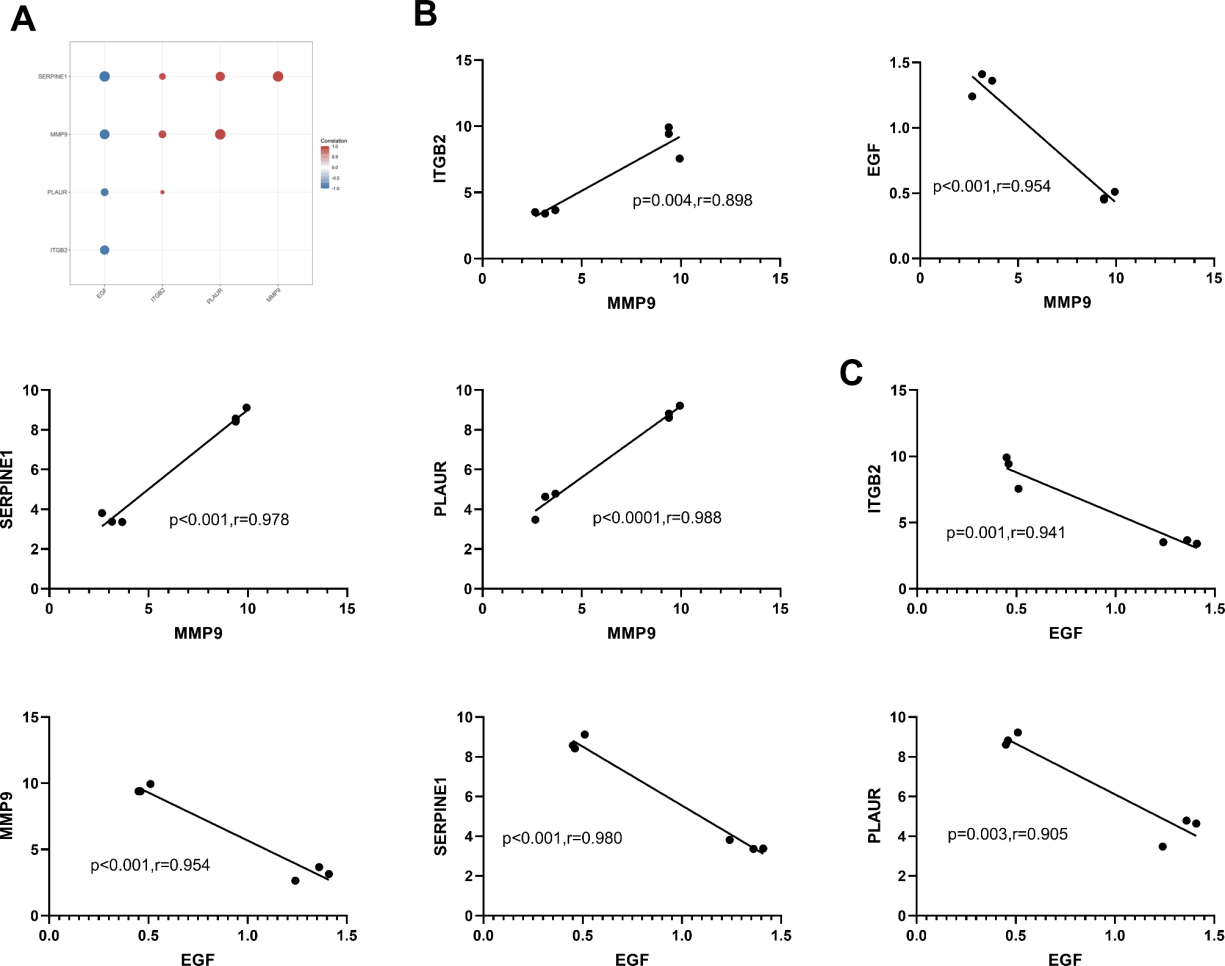
Expression correlation between genes and other central nodes. (**A**) Heat map of the correlation between genes and genes. (**B**) Scatter plot of the expression correlation of MMP9 with other central nodes. (**C**) Scatter plot of the expression correlation of EGF with other central nodes

The expression analysis revealed significant differential expression of *MMP9* and *EGF* between corneal scrapie mice with and without neutrophil closure. Consequently, the correlation between the *MMP9* and *EGF* expressions with other central nodes was investigated. The findings demonstrated a positive correlation between MMP9 expression and *ITGB2*, *SERPINE1*, and *PLAUR* expressions, while *MMP9* was negatively correlated with *EGF* expression. Furthermore, *EGF* expression exhibited a negative correlation with *ITGB2*, *MMP9*, *SERPINE1*, and *PLAUR*, as presented in Fig. 6.

### Immunological analysis

**Fig. 7:**
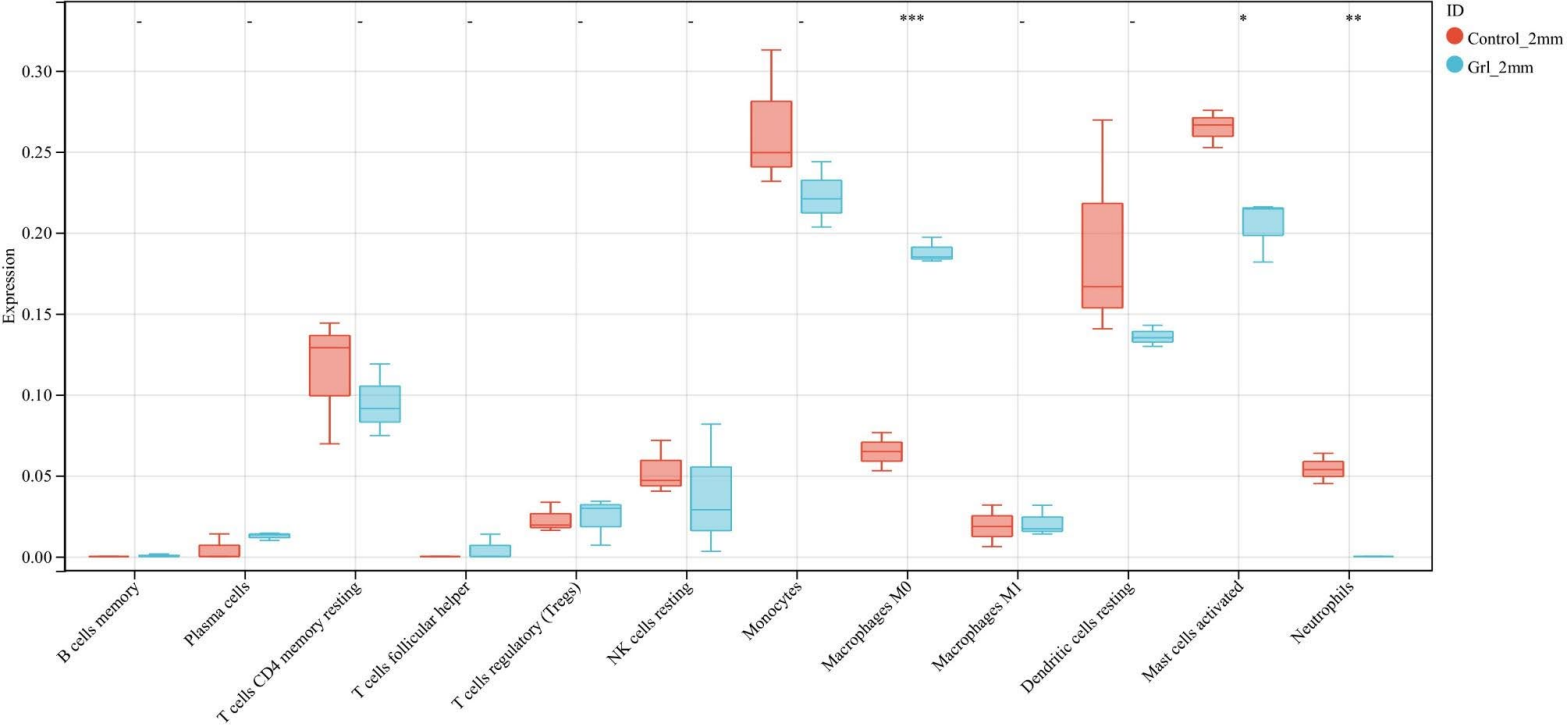
Box plot showing the immune infiltration in both groups

**Fig. 8:**
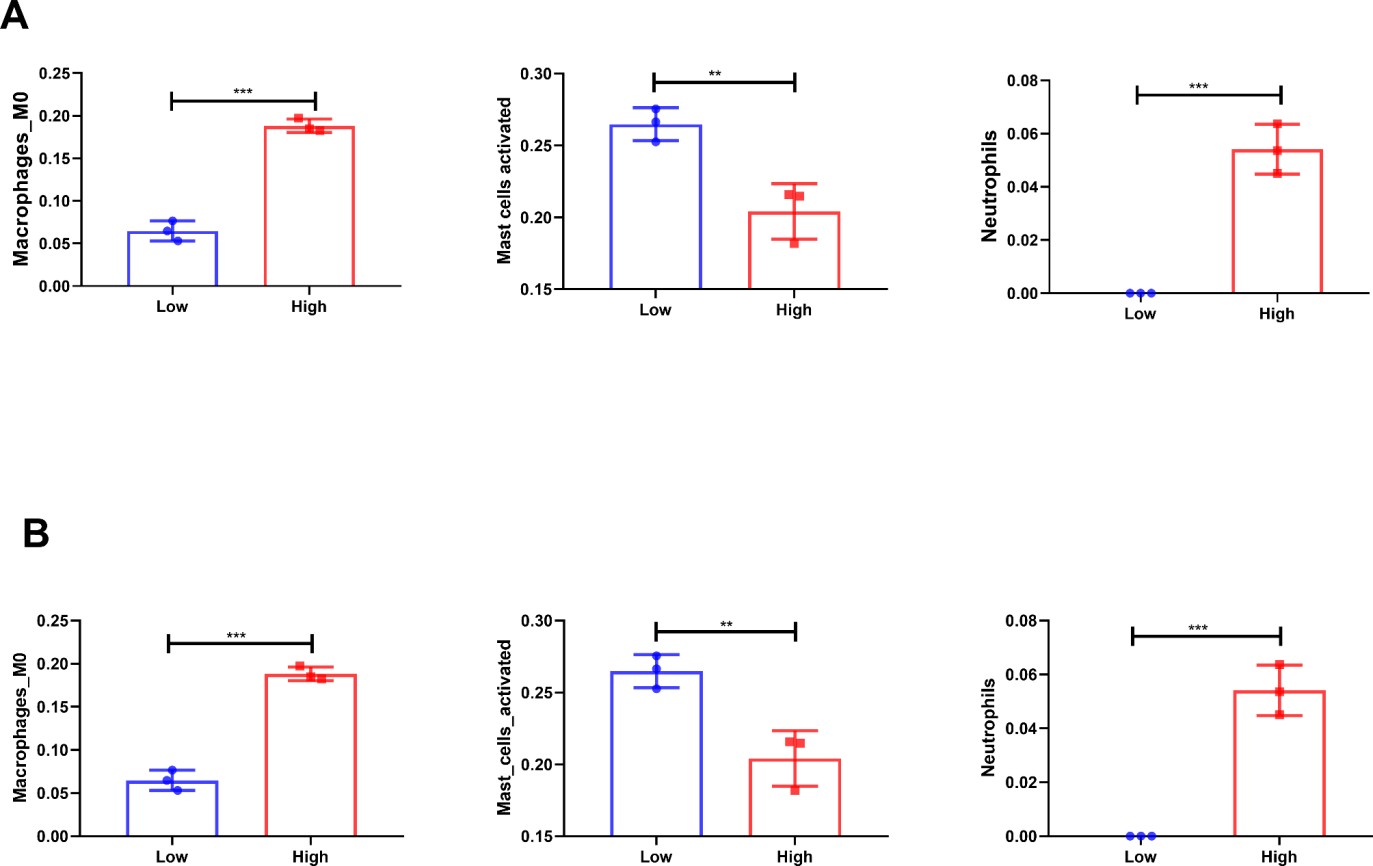
Differences in immune cells between high and low expression groups based on median expression values. (**A**) Differences in M0 macrophages, activated mast cells, and neutrophils between high and low expression groups of MMP9. (**B**) Differences in M0 macrophages, activated mast cells, and neutrophils between high and low expression groups of EGF. **p < 0.01; ***p < 0.001

The immune infiltration between the two groups (with and without neutrophil closure) was further examined. The results revealed significant differences that the infiltration of M0 macrophages, activated mast cells, and neutrophils between the two groups (Fig. 7). Additionally, the samples were classified into high and low-expression groups based on the median values of *MMP9* and *EGF* expressions to observe the differences in immune cells between the two groups. The findings demonstrated that neutrophils and M0 macrophages were higher in the *MMP9* and *EGF* high-expression group, while activated mast cells were lower compared with the *MMP9* and *EGF* low-expression group (Fig. 8).

## Discussion

Neutrophils play a crucial role in an inflammatory response by generating anti-inflammatory and pro-catabolic lipid mediators that aid in the termination of inflammation [11, 12]. Neutrophils can also contribute to pathogenesis through different mechanisms. For instance, at the site of injury, neutrophils release proteases and produce large amounts of reactive oxygen species, leading to tissue damage. This makes the tissue more susceptible to pathogens, thereby promoting the development of chronic inflammation [13]. In cases of corneal damage, neutrophils infiltrate the corneal stroma through the corneal limbal vessels. It is believed that keratinocyte apoptosis facilitates their movement through the stroma toward the wound area. Neutrophil infiltration is a direct response to injury; however, the molecules released by neutrophils, such as oxidative, hydrolytic, and pore-forming molecules, can damage healthy host cells within the injured tissue [14]. Therefore, pathological examination often reveals neutrophil recruitment around the corneal injury [15].

Herein, an analysis of differentially expressed genes in corneal scraping with or without neutrophil closure was conducted. The intersection between these genes and neutrophil-related genes was then identified, resulting in a set of 27 intersected genes. Following this, five key genes (*ITGB2*, *MMP9*, *EGF*, *SERPINE1*, and *PLAUR*) were obtained after the PPI network was constructed using the MCODE algorithm. MMPs constitute a group of zinc endopeptidases crucial for the breakdown of extracellular matrix components and basement membranes. These enzymes play a pivotal role in various physiological and pathological processes, including morphogenesis, wound healing, and inflammation [16]. While *MMP9* is undetectable in healthy tissues, its expression is significantly up-regulated in inflammation or cancer [17]. *MMP9* can impede wound healing by degrading the extracellular matrix associated with normal tissue remodeling [18]. *MMP9* is a crucial enzyme produced by the corneal epithelium that plays a major role in matrix degradation. Its activation is considered a key event in the pathophysiology of corneal ulcers observed in ocular surface diseases. Moreover, it is believed to be a contributing factor in delaying wound healing and re-epithelializing the corneal surface [19]. *EGF* is a naturally occurring mitogen. Upon receptor activation, *EGF* stimulates the synthesis of specific proteins and promotes the proliferation and differentiation of corneal epithelial cells, keratinocytes, and endothelial cells, in in vivo and in vitro settings [20]. Therefore, *EGF* is considered a growth factor that is important for regulating corneal epithelial homeostasis and wound healing. Notably, the topical application of *EGF* has demonstrated the potential to expedite corneal wound healing within hours and enhance the strength of stromal scars, offering possibilities for sutureless surgical approaches [21]. *MMP9* and *EGF* are closely associated with neutrophils. *EGF* has been identified as a crucial factor in epithelial repair and has been shown to enhance the mechanism of neutrophil accumulation, thereby promoting neutrophil defense during acute injuries [22]. Moreover, the major peak of *MMP9* activity is believed to originate from neutrophils [23]. It has also been established that *MMP9* is associated with neutrophil migration, and *MMP9* inhibition significantly hampers the number and rate of neutrophil migration [24]. Our study revealed a significant inhibition of *MMP9* and *EGF* expression in corneal scrapings with neutrophil occlusion in rats. Furthermore, variations in neutrophil levels were observed in samples based on median *MMP9* and *EGF* expression values. The results indicated significantly higher levels of neutrophils in the *MMP9* and *EGF* high-expression group compared with the *MMP9* and *EGF* low-expression group. Previous studies have also indicated the potential significance of epithelial cell-expressed *MMP9* in colitis development, through its involvement in regulating cell-matrix interactions and wound healing [25]. Additionally, research has demonstrated that tumor-associated neutrophil-derived *MMP9* contributes to tumor angiogenesis and progression associated with cutaneous squamous cell carcinoma [26]. Building upon the findings of our study, it can be inferred that corneal injury increases *MMP9* expression [27], and *MMP9* levels can be suppressed by inhibiting neutrophils. This suggests that targeting *MMP9* could hold therapeutic potential and might offer therapeutic benefits.

However, the current study has certain limitations, as the effect of neutrophil closure on nerve regeneration after corneal scraping in mice from a multi-spatial and multi-temporal perspective was not yet examined. Additionally, the effect of *MMP9* inhibition on corneal scraping has not been investigated in vitro. A more in-depth study is warranted in the future.

In summary, through bioinformatics analysis, five key genes associated with neutrophils were identified, among which *MMP9* and *EGF* exhibited significant differential expression between the two groups, with and without neutrophil closure. The *MMP9* and *EGF* expression levels might be closely associated with the neutrophil level.

## Data Availability

The datasets used and/or analyzed during the current study are available from the corresponding author upon reasonable request. The datasets generated and/or analysed during the current study are available in the NCBI repository, http://www.ncbi.nlm.nih.gov/bioproject/937389, SRA accession number: PRJNA937389.

## List of Abbreviations

GO: Gene Ontology
KEGG: Kyoto Encyclopedia of Genes and Genomes
GSEA: Gene set enrichment analysis
ES: Enrichment score
FDR: False-positive discovery rate
NES: Normalized ES
PPI: Protein-protein interaction
MCODE: Molecular Complex Detection
ITGB2: Integrin subunit beta 2
MMP9: Matrix metallopeptidase 9
EGF: Epidermal growth factor
SERPINE1: Serpin family E member 1
PLAUR: Plasminogen activator urokinase receptor
